# Guideline “Motor neuron diseases” of the German Society of Neurology (Deutsche Gesellschaft für Neurologie)

**DOI:** 10.1186/s42466-023-00251-x

**Published:** 2023-06-15

**Authors:** Susanne Petri, Torsten Grehl, Julian Grosskreutz, Martin Hecht, Andreas Hermann, Sarah Jesse, Paul Lingor, Wolfgang Löscher, André Maier, Benedikt Schoser, Marcus Weber, Albert C. Ludolph

**Affiliations:** 1 Klinik für Neurologie, Medizinische Hochschule Hannover, Carl-Neuberg-Str. 1, 30625 Hannover, Germany; 2 Neurologie, Alfried-Krupp-Krankenhaus, Essen, Germany; 3 Neurologie, Universitätsklinikum Lübeck, Lübeck, Germany; 4 Neurologie, Bezirkskrankenhaus Kaufbeuren, Kaufbeuren, Germany; 5 Neurologie, Universitätsmedizin Rostock, Rostock, Germany; 6 Neurologie, Universität Ulm, Ulm, Germany; 7 Neurologie, TU München, Munich, Germany; 8 Neurologie, Medizinische Universität Innsbruck, Innsbruck, Austria; 9 ÖGN, Vienna, Austria; 10 Neurologie, Charité – Universitätsmedizin Berlin, Berlin, Germany; 11 Neurologie, LMU Klinikum München, Munich, Germany; 12 Muskelzentrum, Kantonspital St. Gallen, St. Gallen, Switzerland; 13 SNG, St. Gallen, Switzerland

**Keywords:** Motor neuron disorders, Amyotrophic lateral sclerosis (ALS), Genetics, Neuroprotection, Symptomatic treatment, Interdisciplinary care

## Abstract

**Introduction:**

In 2021, the Deutsche Gesellschaft für Neurology published a new guideline on diagnosis and therapy of motor neuron disorders. Motor neuron disorders affect upper motor neurons in the primary motor cortex and/or lower motor neurons in the brain stem and spinal cord. The most frequent motor neuron disease amyotrophic lateral sclerosis (ALS) is a rapidly progressive disease with an average life expectancy of 2–4 years with a yearly incidence of 3.1/100,000 in Central Europe (Rosenbohm et al. in J Neurol 264(4):749–757, 2017. 10.1007/s00415-017-8413-3). It is considered a rare disease mainly due to its low prevalence as a consequence of short disease duration.

**Recommendations:**

These guidelines comprise recommendations regarding differential diagnosis, neuroprotective therapies and multidisciplinary palliative care including management of respiration and nutrition as well as provision of assistive devices and end-of-life situations.

**Conclusion:**

Diagnostic and therapeutic guidelines are necessary due the comparatively high number of cases and the aggressive disease course. Given the low prevalence and the severe impairment of patients, it is often impossible to generate evidence-based data so that ALS guidelines are partially dependent on expert opinion.

## Introduction

This guideline is an abridged and translated short version of “Motoneuronerkrankungen”.

A complete version of this guideline (in German) can be found on the website of the Deutsche Gesellschaft für Neurologie (www.dgn.org/leitlinien) and the AWMF (Arbeitsgemeinschaft wissenschaftlicher Medizinischer Gesellschaften) [34].

- *AWMF Registry number:* 8.0a.
- *Level of guideline:* S1.
- *Date of last update:* 11/2021.
- *Valid until:* 12.08.2026.
- *Edited by:* Guidelines Committee of the German Neurological Society.

ALS is a rapidly progressive multisystem degenerative disease with predominant motor symptoms. Involvement of extramotor brain regions is frequent and has been confirmed by neuropsychological, electrophysiological and neuropathological as well as imaging studies [8, 19, 22, 29, 54]. Different clinical phenotypes are defined by the region of disease onset (bulbar/cervical/thoracic/lumbar) and the extent of upper and lower motor neuron and extramotor involvement. Up to 5% of ALS patients develop clinically manifest frontotemporal dementia (ALS/FTD) while more subtle frontal deficits can occur in up to 50% of patients [37]. Spinal muscular atrophy (SMA) can be diagnosed by genetic testing for mutations in the survival of motor neuron gene on chromosome 5 and mostly becomes apparent with lower motor neuron signs in childhood or adolescence. Only slower progressive adult onset forms (SMA type 4) can show clinical overlap with ALS in the variant of progressive muscular atrophy (PMA) [61]. The occurence of signs of combined affection of bulbar and spinal motor neurons without upper motor neuron symptoms in male patients is suggestive for x-chromosomal recessive spinobulbar muscular atrophy (SBMA, Kennedy’s syndrome) which is caused by expansion of a tandem CAG repeat in the first exon of the androgen receptor gene on chromosome Xq11-12. Motor symptoms frequently are combined with postural tremor, fasciculations and endocrinological symptoms (gynecomastia, testicular atrophy, oligospermia, diabetes) [31]. Hereditary spastic paraplegia (HSP) can be confounded with the ALS variant primary lateral sclerosis (PLS) as both disorders lead to progressive upper motor neuron damage (with HSP being rather symmetrical as opposed to a focal onset in PLS).

The most important etiological factor of ALS are mutations in the C9ORF72 gene accounting for about 25% of familial and up to 10% of sporadic cases in Europe [44]. Intermediate length polyglutamine expansions in the ataxin-2-gene have been identified as most important genetic risk factor for sporadic ALS [17]. Mutations in the gene coding for cytosolic Cu/Zn superoxide dismutase (Cu/Zn SOD) account for 10–15% of autosomal-dominantly inherited cases in Germany. Autosomal-dominant mutations in the fused in sarcoma (FUS) (also in very young patients) and TARDNP (TDP-43)-genes are responsible for less than 5% [17]. Penetrance can be incomplete, in particular in C9ORF72-mutations, which implies that these mutations can also occur in apparently sporadic cases [27].

## What’s new?

- Recent data support the concept of genetic, pathological and pathophysiological overlaps within the spectrum of degenerative motor neuron disorders consisting of amyotrophic lateral sclerosis (ALS) associated with aggregation of three specific proteins (TDP-43, SOD1, FUS), the genetically heterogeneous hereditary spastic paraplegias (HSP), and the more homogenous spinobulbar muscular atrophy (SBMA) and spinal muscular atrophy (SMA)
- The relationship between ALS and frontotemporal dementia (FTD) is well established and of both diagnostic and therapeutic relevance
- The ALS spectrum includes distinct clinical phenotypes with variable degrees of upper and lower motor neuron as well as extramotor (mainly frontotemporal) involvement such as primary lateral sclerosis (PLS), flail arm-/flail leg-syndrome, progressive muscular atrophy (PMA), progressive bulbar palsy (PBP), and ALS/frontotemporal dementia (FTD)
- Criteria (Brettschneider-Braak-stages) can be used for neuropathological subclassification and staging. Although these stages are mirrored by imaging, neuropsychological, and neurophysiological studies, their clinical use is restricted to an inproved understanding of the pattern of progression and pareses. A preclinical stage of ALS has not been defined
- Measurement of neurofilament concentrations, in particular of neurofilament light chain (NfL) in serum and phosphorylated neurofilament heavy chain (pNfH) in CSF is, while not ALS-specific, useful as diagnostic and prognostic marker
- Intravenous administration of the antioxidant edaravone has been approved for treatment of ALS in Japan, the US, Canada, Switzerland and South Corea. Phase III-studies with orally administered edaravone are currently under way also in Europe
- Positive effects in ALS patients with rapid disease progression could be shown for the MAO-B-inhibitor rasagiline and for a high caloric fatty diet in post hoc analyses of phase IIb-studies but final conclusions cannot be drawn yet
- Treatment with the antisense oligonucleotide nusinersen has been approved in Germany for treatment of patients with spinal muscular atrophy with mutations in chromosome 5q (5q-SMA) since 2017 without age restrictions. First real world data show stabilization and/or improvement of symptoms in adult SMA patients. An alternative for adult SMA patients is the oral splicing modifier risdiplam
- Antisense oligonucleotide-based treatments are in clinical development also for several genetic forms of ALS

## The most important recommendations

- Riluzole (2 × 50 mg/d) delays disease progression
- Aims of symptomatic (palliative) therapy are maintenance of quality of life and autonomy, early information about the disease and preparation of advance directives
- Early information of patients and caregivers about non-invasive ventilation as an effective symptomatic and survival-prolonging treatment is important
- Pneumonia should be prevented by physical therapy and early antibiotic treatment of respiratory tract infections as well as treatment of sialorrhea
- Percutaneous endoscopic gastrostomy (PEG) can prevent catabolism and is probably associated with increased survival, in particular in combination with specific nutritional strategies and improvement of quality of life
- Symptomatic treatment of dysarthria and other disease-associated symptoms (pseudosioalorrhea, thick mucus, depression, cramps, pain) is recommended
- Adequate provision of assistive devices for maintenance of autonomy and prevention of secondary complications should be initiated early
- Advance directives should be created and regularly reviewed and access to palliative care should be initiated early enough

## Guidelines in detail

### Diagnosis

The diagnosis of ALS is primarily based on clinical criteria. Differential diagnosis can be difficult in early disease stages and if signs of upper or—less frequently—lower motor neuron affection are lacking.

Distinct clinical phenotypes can be distinguished:

- Classical ALS (upper and lower motor neuron signs with bulbar or spinal onset)
- Upper motor neuron-predominant ALS
- Primary lateral sclerosis (PLS) as pure upper motor neuron syndrome which may only affect bulbar muscles (pseudo-bulbar palsy) [58]
- Flail arm-/flail leg-syndrome, leading in most patients initially to an often proximally predominant paraparesis of upper or lower extremities without abnormalities in DTR
- Progressive bulbar palsy (PBP)
- Axial variant with primary hypoventilation and weakness of trunk muscles (“thoracic onset”)
- Progressive muscular atrophy (PMA) with lack of clinically apparent upper motor neuron signs
- ALS/FTD (5–10% of patients) combining motor symptoms and behavioral/cognitive deficits typical of FTD

While the El Escorial diagnostic criteria (last revised in 2000) requested the presence of both upper and lower motor neuron signs in at least on body region for a diagnosis of “possible ALS” [10], a revision has been initiated in 2015 by the Word Federation of Neurology aiming to better integrate recent knowledge and specific phenotypes [35]. In line with these suggestions, an international group of experts published the so-called Gold Coast criteria in 2020 [55]. Using these criteria, progressive motor impairment with either upper and lower motor neuron affection in one or lower motor neuron affection in at least two body regions can be diagnosed as ALS if other alternative disease processes are excluded by appropriate examinations. These suggested criteria are sensitive for the diagnosis, but their specificity is not explored yet.

The revised version of the ALS Functional Rating Scale (ALSFRSr) is an easily applicable clinical score which can reflect disease progression despite some methodological limitations. It can be used for patient care as well as an outcome parameter in clinical trials and also as patient reported outcomes by patients themselves. Printed and only versions are available [38].

### Diagnostics

#### Mandatory examinations

Clinical-neurological examination taking into account the typical patters (“central”, “corticospinal”) of muscle weakness and disease spreading.

Electromyography and nerve conduction studies (including assessment of potential conduction blocks).

Cranial and spinal MRT based on the clinical presentation (e.g. to exclude myelopathy/polyradicular lesions).

Neuropsychological testing, if possible including the Edinburgh Cognitive and Behavioral ALS Screen (ECAS).

Pulmonary function tests (vital capacity, SNIP), nocturnal capnometry during disease progression.

Body weight, body mass index (BMI).

Regular recording of the ALSFRSR.

#### Additional tests in according to the clinical and neurophysiological findings and resulting differential diagnoses

CSF analysis (lymphocyte count, protein content, oligoclonal bands, glucose, lactate, neurofilament concentrations (NfL, pNfH in serum and CSF)).

Blood sedimentation rate, C-reactive protein (CRP), red, white, differentia blood count, GOT, GPT, TSH, T3,T4, vitamine B12 (methylmalonic acid, homocysteine), serum- and immune electrophoresis, CK, creatinine, Na^+^, K^+^, Ca^2+^, Cl^−^, PO_4_, glucose.

#### Additional laboratory examinations in individual cases:

- Angiotensin converting enzyme/ACE, Hexosaminidase A and B
- Antibody testing for differential diagnosis of immune-mediated diseases (e.g. ANA, antiDNA, Anti-Hu, anti-MAG, Anti-AchR, anti-MUSK, potassium channel antibodies, antibodies against voltage-gated calcium channels, MUSK-, acetylcholine receptor-antibodies)
- In cases with dementia: VLCFA (very long chain fatty acids) in Serum, Arylsulfatase A
- In cases with myogenic changes at the EMG examination: muscle biopsy (for differential diagnosis of inclusion body myositis/distal myopathies/glycogen-storage diseases)
- Examination of swallowing including FEES, HNT examination in cases with isolated bulbar/pseudobulbar symptoms

### Genetic testing

Genetic testing (currently as panel diagnostics and additional testing for C9ORF72 repeat expansions with Southern Blot for determination of repeat length) should routinely be performed only in patients with positive family history (including dementias and psychiatric disorders) after obtaining informed consent.

These recommendations may change in case of available disease modifying gene therapies (particularly regarding SOD1, C9ORF72 and FUS mutations) rendering genetic testing relevant also for apparently sporadic cases (due to lack of complete penetrance of some mutations and the possibility of de-novo-mutations).

Testing for mutations in the androgen receptor and SMN1 genes should be initiated in cases with isolated lower motor neuron affection leading to clinical suspicion of SBMA or SMA.

## Therapy

### Disease modifying pharmacotherapy

There is a high unmet need for more efficient neuroprotective treatments of ALS. So far, only riluzol has been approved in Europe as disease modifying treatment of ALS. Its acts as a blocker of voltage-gated sodium channels and has been shown to significantly increase survival without tracheostomy after 12 months [43]. Pooled data from clinical registries showed a survival increase of up to 10 months [4, 26]. The comparison of different doses showed best efficacy with best tolerability of a dose of 100 mg/d [32]. It is available as tablet or suspension and in general well tolerated but can lead to a significant increase in liver enzymes.

The antioxidant edaravone has so far been approved for the treatment of ALS in the US, Canada, South Korea and Switzerland. A randomized placebo-controlled trial had not shown efficacy in a mixed ALS patient population [1] but a subsequent study with inclusion criteria based on post hoc analyses showed significant slowing of deterioration in the ALSFRS-R in selected patients while data on survival are lacking so far [62]. Phase 3 studies with oral edaravone formulations as well as other experimental treatments are currently ongoing.

All ALS patients should be treated with riluzol in a dosage of 50 mg BID, transaminases should be regularly monitored. A general recommendation for edaravone can not be given at the moment. Patients should be offered the possibility to participate in clinical trials in specialized MND clinics.

### Physical and occupational therapy

Aims of physical therapy are maintenance of functionality and autonomy, quality of life, cardiovascular function and prevention of secondary complications such as pain and contractures [6, 14, 30]. Measures such as lymphatic drainage or respiratory therapy should be initiated according to patients’ symptoms.

Speech and swallowing therapy should be started early in patients with bulbar affection and should be complemented by advice on communication devices with further disease progression [51].

### Chronic respiratory insufficiency

Non-invasive ventilation (NIV) can increase survival and improve quality of life of ALS patients [7, 45, 49]. Patients with predominantly spinal as opposed to bulbar symptoms show better benefit in general. Pulmonary function should be regularly monitored and patients should be informed early about treatment options and limitations.

NIV should be started when symptoms of chronic hypoventilation become apparent or increased pCO2 levels are detected at nocturnal capnometry.

Mechanic insufflator/exsufflator (cough assist) devices can used to alleviate weak cough and bronchial obstruction by thick mucus.

Diaphragm pacing cannot be recommended [23, 40].

Prior to tracheostomy and invasive ventilation, the individual disease course, set of values and psychosocial resources of each patient should be considered.

### Prevention of pneumonia

Pneumonia should be prevented by physical therapy, inhibition of mucus production (if necessary by gastrostomy), treatment of sialorrhea and use of mechanic insufflators/exsufflators [53].

### Palliative care/terminal respiratory insufficiency

Individual treatment aims and limitations including non-invasive and invasive ventilation and percutaneous endoscopic gastrostomy (PEG) should be discussed early and repeatedly with patients and caregivers.

In cases of respiratory distress, inhalation with bronchodilators such as ipratropiumbromide/salbutamole), morphine (s.c./i.v./nasal spray) and anxiolytics (e.g. lorazepam, midazolam) can be used.

### Sialorrhea

Sialorrhea as a result of impaired swallowing should be treated to prevent social stigmatization and pneumonia. Pharmacological treatment options include pirenzepine, ipratropium bromide spray, scopolamine transdermal patch, amitriptyline, sublingual application of atropine 1% eye drops, ultrasound-guided injection of incobotulinumtoxin A into the parotid and submandibular salivary glands, starting with 100 MU (approved for the treatment of sialorrhea in 2019 [28]).

Careful dose titration is important to prevent dry mouth [63].

Fractionated radiation of salivary glands (7–8 Gray) is an option in treatment-resistant sialorrhea [5].

### Laryngospasms

Laryngospasms are frequent in SBMA but may also occur in ALS partients [20]. Patients should be informed that they are harmless and self limitating. If patients are suffering from them, treatment can be attempted by proton pump inhibitors or prokinetic agents, in a second step by botulinum toxin injections into the vocal folds [18].

### Prevention of thrombosis

Risk for deep vein thrombosis in ALS is highly variable and depends on the location and severity of pareses and presence of other risk factors.

As there are no larger studies in ALS patients, recommendations should be based on general recommendations for paraplegic patients [59].

In addition to basic measures such as sufficient hydration and mobilization by physical therapy, severely immobilized patients should receive prophylactic treatment with low molecular heparins. In case of contra-indications, direct oral anticoagulants (DOAK) in reduces dosages may be used off-label [64].

### Treatment of dysphagia and catabolism

Nutritional status of ALS patients is an independent risk factor for survival [16]. Dysphagia occurs early in bulbar onset ALS and frequently also in spinal onset patients within the disease course. FEES may be helpful to detect clinically inapparent dysphagia and for adjustment of logopedic treatment [46]. Catabolism can be caused not only by dysphagia but also due to respiratory insufficiency, hypermetoabolism or loss of appetite. Positive effects of a high caloric diet could not be confirmed in a clinical phase 2b trial for the whole study population but post hoc analyses indiated beneficial effects particularly in patients with rapid disease progression [36].

Patients should be regularly monitored for dysphagia and weight loss. Treatment should be initiated in cases of patients’ suffering, weight loss, dehydration and risk of aspiration. First measures should be nutrition counselling and prescription of high caloric liquid nutrition as well as logopedic treatment. Percutaneous endoscopic gastrostomy (PEG) with pre-intervention antibiotic treatment, slow restoration of feeding and peri-interventional ventilation if required is recommended in cases of more advanced dysphagia and weight loss.

### Dysarthria

Early initiation of measures facilitating patients communication abilities can improve patients’ and caregivers quality of life [33].

The combination of quinidine/dextromethorphan had positive effects on bulbar function in a short randomized trial [57] and may be prescribed by specialized clinics [41]. Measures to improve communication include speech therapy, alphabet tables, communication apps on tablets/mobile phones and electronic speech devices. Brain-computer interfaces have so far only been tested in experimental settings and their use in ALS may be limited by multisystem neurodegeneration in advanced stages.

### Depression

Patients with ALS are at higher risk to develop depression and to commit suicide than the general population [52, 56]. This is particularly true for early stages of the disease and in patients with a history of affective disorders and/or a insufficient social support.

There is a lack of large and well-designed trials to assess pharmacological interventions against depression in ALS patients [21, 24]. ALS patients with clinically manifest depression should therefore be treated according to general national and international depression guidelines taking into account other ALS related symptoms: anticholinergic side effects may be useful in ALS patients with sialorrhea, antinociceptive effects in patients with pain, sedation and increase of appetite in patients with sleep disturbances and weight loss.

### Emotional lability

Increased emotional lability and pathological laughing/crying are often more disturbing for relatives than for patients themselves.

Patients should be informed about emotional lability as a symptom of ALS. Selective serotonin reuptake inhibitors such as citalopram or tricyclics such as amitriptyline can be used off-label to treat emotional lability. A small clinical trial showed efficacy of quinidin/dextrometrophan but with frequent side effects [11].

### Pain

Pain is a frequently underdiagnosed symptom in ALS [13]. It should be classified and treated according to pain type (nociceptive, musculoskeletal, neuropathic) based on national/international guidelines.

### Muscle cramps

Fasciculations and cramps are a frequent and mostly temporary symptom mainly in early disease stages. Fasciculations only very rarely require treatment. Cramps can be treated with magnesium, quinidine sulfate, mexiletine (of which the efficacy has been confirmed in two randomized clinical trials in ALS patients [47, 60]) or ranozaline. In treatment resistant cases, cannabinoids may be used.

### Spasticity

Spastiticy resulting in contractures and musculoskeletal pain primarily occurs in patients with upper motor neuron dominant ALS or PLS and can be treated with physical therapy and antispastic agents such as lioresal.

Delta-9-tetrahydrocannabinol and cannabidiol (THC/CBD: 50:50) spray resulted in decreased spasticity symptoms in a placebo-controlled randomized phase 2 trial in ALS patients [50]. Botulinum toxin A may be used in focal treatment resistant spasticity [39].

### Anxiety

Data on the prevalence of anxiety in ALS patients are sparse but smaller studies indicate that it is not more frequent than in the general population [25, 48].

As no large well-designed clinical trials on the treatment of anxiety in ALS exist, it should be performed according to general guidelines. Depending on the severity, psychotherapeutic and/or pharmacological treatments should be used, including short acting benzodiazepines for acute attacks and antidepressants (SSRI, SNRI, tricyclics) in chronic anxiety.

### Assistive devices

Provision of assistive devices to maintain patients’ autonomy, communication and mobility, enable social participation and symptom control is a central part in the treatment of ALS, It should preferably occur in specialized centers experienced in the care of ALS patients.

### Psychosocial care

Psychosocial care should be offered to patients and caregivers and information on patient support groups should be provided by specialized MND clinics. It may contribute to alleviate the burden of caregivers [12, 15].

### Coordination of care

Diagnosis of ALS should preferable made during an inpatient stay in the hospital. In cases seeking for a second opinion, an ambulatory setting in specialized MND clinics can be an alternative. Further treatment of ALS patients including coordination of symptomatic treatment measures and care as well as advance care planning should also take place in an interdisciplinary ALS center. Individual treatment aims and limitations should be discussed and the option to create advanced directives should be offered. Specialized outpatient palliative care should be organized if needed.

In cases with more complex or life-threatening symptoms, inpatient hospital admission is required for symptom control. Patients with hypoventilation and the need for non-invasive/invasive ventilation should be treated in specialized centers who are experienced in the treatment of neuromuscular diseases. PEG placement should also be performed in an inpatient setting, experienced centers are strongly recommended.

For symptom control in patients with terminal respiratory insufficiency, inpatient treatment in palliative care wards or hospices can be considered.

## References

[1] Abe K, Itoyama Y, Sobue G, Tsuji S, Aoki M, Doyu M, Edaravone ALS Study Group (2014). Confirmatory double-blind, parallel-group, placebo-controlled study of efficacy and safety of edaravone (MCI-186) in amyotrophic lateral sclerosis patients. Amyotrophic Lateral Sclerosis Frontotemporal Degeneration.

[2] Andersen PM, Abrahams S, Borasio GD, de Carvalho M, Chio A, Van Damme P, Weber M (2012). EFNS guidelines on the clinical management of amyotrophic lateral sclerosis (MALS)—Revised report of an EFNS task force. European Journal of Neurology.

[3] Andersen PM, Borasio GD, Dengler R, Hardiman O, Kollewe K, Leigh PN, Management of Amyotrophic Lateral, S. (2005). EFNS task force on management of amyotrophic lateral sclerosis: Guidelines for diagnosing and clinical care of patients and relatives. European Journal of Neurology.

[4] Andrews JA, Jackson CE, Heiman-Patterson TD, Bettica P, Brooks BR, Pioro EP (2020). Real-world evidence of riluzole effectiveness in treating amyotrophic lateral sclerosis. Amyotroph Lateral Scler Frontotemporal Degener.

[5] Assouline A, Levy A, Abdelnour-Mallet M, Gonzalez-Bermejo J, Lenglet T, Le Forestier N, Pradat PF (2014). Radiation therapy for hypersalivation: A prospective study in 50 amyotrophic lateral sclerosis patients. International Journal of Radiation Oncology Biology Physics.

[6] Bello-Haas VD, Florence JM, Kloos AD, Scheirbecker J, Lopate G, Hayes SM, Mitsumoto H (2007). A randomized controlled trial of resistance exercise in individuals with ALS. Neurology.

[7] Bourke SC, Tomlinson M, Williams TL, Bullock RE, Shaw PJ, Gibson GJ (2006). Effects of non-invasive ventilation on survival and quality of life in patients with amyotrophic lateral sclerosis: A randomised controlled trial. Lancet Neurology.

[8] Braak H, Brettschneider J, Ludolph AC, Lee VM, Trojanowski JQ, Del Tredici K (2013). Amyotrophic lateral sclerosis—A model of corticofugal axonal spread. Nature Reviews Neurology.

[9] Bradley WG, Anderson F, Bromberg M, Gutmann L, Harati Y, Ross M, Group, A. C. S. (2001). Current management of ALS: Comparison of the ALS CARE database and the AAN practice parameter. The American Academy of Neurology. Neurology.

[10] Brooks BR, Miller RG, Swash M, Munsat TL, World Federation of Neurology Research Group on Motor Neuron, D. (2000). El Escorial revisited: Revised criteria for the diagnosis of amyotrophic lateral sclerosis. Amyotroph Lateral Sclerosis Other Motor Neuron Disorder.

[11] Brooks BR, Thisted RA, Appel SH, Bradley WG, Olney RK, Berg JE, Group, A.-A. S. (2004). Treatment of pseudobulbar affect in ALS with dextromethorphan/quinidine: a randomized trial. Neurology.

[12] Burke T, Elamin M, Galvin M, Hardiman O, Pender N (2015). Caregiver burden in amyotrophic lateral sclerosis: A cross-sectional investigation of predictors. Journal of Neurology.

[13] Chio A, Mora G, Lauria G (2017). Pain in amyotrophic lateral sclerosis. Lancet Neurology.

[14] de Almeida JP, Silvestre R, Pinto AC, de Carvalho M (2012). Exercise and amyotrophic lateral sclerosis. Neurological Sciences.

[15] de Wit J, Bakker LA, van Groenestijn AC, van den Berg LH, Schroder CD, Visser-Meily JMA, Beelen A (2018). Caregiver burden in amyotrophic lateral sclerosis: A systematic review. Palliative Medicine.

[16] Dupuis L, Pradat PF, Ludolph AC, Loeffler JP (2011). Energy metabolism in amyotrophic lateral sclerosis. Lancet Neurology.

[17] Elden AC, Kim HJ, Hart MP, Chen-Plotkin AS, Johnson BS, Fang X, Gitler AD (2010). Ataxin-2 intermediate-length polyglutamine expansions are associated with increased risk for ALS. Nature.

[18] Faham M, Ahmadi A, Silverman E, Harouni GG, Dabirmoghaddam P (2021). Quality of life after botulinum toxin injection in patients with adductor spasmodic dysphonia: A systematic review and meta-analysis. Journal of Voice.

[19] Finegan E, Hi Shing SL, Chipika RH, McKenna MC, Doherty MA, Hengeveld JC, Bede P (2020). Thalamic, hippocampal and basal ganglia pathology in primary lateral sclerosis and amyotrophic lateral sclerosis: Evidence from quantitative imaging data. Data in Brief.

[20] Gdynia HJ, Sperfeld AD, Flaith L, Kuehnlein P, Unrath A, Ludolph AC, Kassubek J (2007). Classification of phenotype characteristics in adult-onset spinal muscular atrophy. European Neurology.

[21] Gnanapragasam S, Hopkins CW, Moulton CD (2016). Can pharmacotherapy improve depressive symptoms in patients with amyotrophic lateral sclerosis? A systematic review of the literature. Amyotroph Lateral Scler Frontotemporal Degener.

[22] Goldstein LH, Abrahams S (2013). Changes in cognition and behaviour in amyotrophic lateral sclerosis: Nature of impairment and implications for assessment. Lancet Neurology.

[23] Gonzalez-Bermejo J, Morelot-Panzini C, Tanguy ML, Meininger V, Pradat PF, Lenglet T, Similowski T (2016). Early diaphragm pacing in patients with amyotrophic lateral sclerosis (RespiStimALS): A randomised controlled triple-blind trial. Lancet Neurology.

[24] Gould RL, Coulson MC, Brown RG, Goldstein LH, Al-Chalabi A, Howard RJ (2015). Psychotherapy and pharmacotherapy interventions to reduce distress or improve well-being in people with amyotrophic lateral sclerosis: A systematic review. Amyotroph Lateral Scler Frontotemporal Degener.

[25] Grabler MR, Weyen U, Juckel G, Tegenthoff M, Mavrogiorgou-Juckel P (2018). Death anxiety and depression in amyotrophic lateral sclerosis patients and their primary caregivers. Frontiers in Neurology.

[26] Hinchcliffe M, Smith A (2017). Riluzole: Real-world evidence supports significant extension of median survival times in patients with amyotrophic lateral sclerosis. Degenerative Neurological and Neuromuscular Disease.

[27] Hubers A, Weishaupt JH, Ludolph AC (2013). Genetics of amyotrophic lateral sclerosis. Der Nervenarzt.

[28] Jost WH, Friedman A, Michel O, Oehlwein C, Slawek J, Bogucki A, Blitzer A (2019). SIAXI: Placebo-controlled, randomized, double-blind study of incobotulinumtoxinA for sialorrhea. Neurology.

[29] Kassubek J, Muller HP, Del Tredici K, Brettschneider J, Pinkhardt EH, Lule D, Ludolph AC (2014). Diffusion tensor imaging analysis of sequential spreading of disease in amyotrophic lateral sclerosis confirms patterns of TDP-43 pathology. Brain.

[30] Kato N, Hashida G, Konaka K (2018). Effect of muscle strengthening exercise and time since onset in patients with amyotrophic lateral sclerosis: A 2-patient case series study. Medicine (Baltimore).

[31] Kuhlenbaumer G, Bocchicchio M, Kress W, Young P, Oberwittler C, Stogbauer F (1998). X-chromosomal recessive spinobulbar muscular atrophy (Kennedy type). Description of a family, clinical aspects, molecular genetics, differential diagnosis and therapy. Der Nervenarzt.

[32] Lacomblez L, Bensimon G, Leigh PN, Guillet P, Meininger V (1996). Dose-ranging study of riluzole in amyotrophic lateral sclerosis. Amyotrophic Lateral Sclerosis/Riluzole Study Group II. Lancet.

[33] Londral A, Pinto A, Pinto S, Azevedo L, De Carvalho M (2015). Quality of life in amyotrophic lateral sclerosis patients and caregivers: Impact of assistive communication from early stages. Muscle and Nerve.

[34] Ludolph, A., Petri, S., Grosskreutz, J. et al. (2021). Motoneuronerkrankungen, S1-Leitlinie. In Deutsche Gesellschaft für Neurologie (Hrsg.), Leitlinien für Diagnostik und Therapie in der Neurologie. www.dgn.org/leitlinien

[35] Ludolph A, Drory V, Hardiman O, Nakano I, Ravits J, Robberecht W, WFN Research Group On ALS/MND (2015). A revision of the El Escorial criteria—2015. Amyotroph Lateral Scler Frontotemporal Degener.

[36] Ludolph AC, Dorst J, Dreyhaupt J, Weishaupt JH, Kassubek J, Weiland U, Group, L.-A. S. (2020). Effect of high-caloric nutrition on survival in amyotrophic lateral sclerosis. Annals of Neurology.

[37] Lule DE, Aho-Ozhan HEA, Vazquez C, Weiland U, Weishaupt JH, Otto M, Ludolph AC (2019). Story of the ALS-FTD continuum retold: Rather two distinct entities. Journal of Neurology, Neurosurgery and Psychiatry.

[38] Maier A, Holm T, Wicks P, Steinfurth L, Linke P, Munch C, Meyer T (2012). Online assessment of ALS functional rating scale compares well to in-clinic evaluation: A prospective trial. Amyotrophic Lateral Sclerosis.

[39] Marvulli R, Megna M, Citraro A, Vacca E, Napolitano M, Gallo G, Ianieri G (2019). Botulinum toxin type A and physiotherapy in spasticity of the lower limbs due to amyotrophic lateral sclerosis. Toxins (Basel).

[40] McDermott CJ, Bradburn MJ, Maguire C, Cooper CL, Baird WO, Baxter SK, Shaw PJ (2016). DiPALS: Diaphragm pacing in patients with amyotrophic lateral sclerosis—A randomised controlled trial. Health Technology Assessment.

[41] Meyer T, Kettemann D, Maier A, Grehl T, Weyen U, Grosskreutz J, Spittel S (2020). Symptomatic pharmacotherapy in ALS: Data analysis from a platform-based medication management programme. Journal of Neurology, Neurosurgery and Psychiatry.

[42] Miller RG, Jackson CE, Kasarskis EJ, England JD, Forshew D, Johnston W, Quality Standards Subcommittee of the American Academy of, N. (2009). Practice parameter update: The care of the patient with amyotrophic lateral sclerosis: Multidisciplinary care, symptom management, and cognitive/behavioral impairment (an evidence-based review): report of the Quality Standards Subcommittee of the American Academy of Neurology. Neurology.

[43] Miller RG, Mitchell JD, Moore DH (2012). Riluzole for amyotrophic lateral sclerosis (ALS)/motor neuron disease (MND). Cochrane Database Systematic Review.

[44] Muller K, Brenner D, Weydt P, Meyer T, Grehl T, Petri S (2018). Comprehensive analysis of the mutation spectrum in 301 German ALS families. Journal of Neurology, Neurosurgery and Psychiatry.

[45] Mustfa N, Walsh E, Bryant V, Lyall RA, Addington-Hall J, Goldstein LH, Leigh PN (2006). The effect of noninvasive ventilation on ALS patients and their caregivers. Neurology.

[46] Onesti E, Schettino I, Gori MC, Frasca V, Ceccanti M, Cambieri C, Inghilleri M (2017). Dysphagia in amyotrophic lateral sclerosis: Impact on patient behavior, diet adaptation, and Riluzole management. Frontiers in Neurology.

[47] Oskarsson B, Moore D, Mozaffar T, Ravits J, Wiedau-Pazos M, Parziale N, McDonald CM (2018). Mexiletine for muscle cramps in amyotrophic lateral sclerosis: A randomized, double-blind crossover trial. Muscle and Nerve.

[48] Pagnini F, Marconi A, Tagliaferri A, Manzoni GM, Gatto R, Fabiani V, Lunetta C (2017). Meditation training for people with amyotrophic lateral sclerosis: A randomized clinical trial. European Journal of Neurology.

[49] Radunovic A, Annane D, Rafiq MK, Brassington R, Mustfa N (2017). Mechanical ventilation for amyotrophic lateral sclerosis/motor neuron disease. Cochrane Database Systematic Review.

[50] Riva N, Mora G, Soraru G, Lunetta C, Ferraro OE, Falzone Y, Group, C. S. (2019). Safety and efficacy of nabiximols on spasticity symptoms in patients with motor neuron disease (CANALS): A multicentre, double-blind, randomised, placebo-controlled, phase 2 trial. The Lancet Neurology.

[51] Rogus-Pulia NM, Plowman EK (2020). Shifting tides toward a proactive patient-centered approach in dysphagia management of neurodegenerative disease. American Journal of Speech-Language Pathology.

[52] Roos E, Mariosa D, Ingre C, Lundholm C, Wirdefeldt K, Roos PM, Fang F (2016). Depression in amyotrophic lateral sclerosis. Neurology.

[53] Sancho J, Servera E, Diaz J, Marin J (2004). Efficacy of mechanical insufflation-exsufflation in medically stable patients with amyotrophic lateral sclerosis. Chest.

[54] Seer C, Joop M, Lange F, Lange C, Dengler R, Petri S, Kopp B (2017). Attenuated error-related potentials in amyotrophic lateral sclerosis with executive dysfunctions. Clinical Neurophysiology.

[55] Shefner JM, Al-Chalabi A, Baker MR, Cui LY, de Carvalho M, Eisen A, Kiernan MC (2020). A proposal for new diagnostic criteria for ALS. Clinical Neurophysiology.

[56] Silva-Moraes MH, Bispo-Torres AC, Barouh JL, Lucena PH, Armani-Franceschi G, Dorea-Bandeira I, Bandeira ID (2020). Suicidal behavior in individuals with amyotrophic lateral sclerosis: A systematic review. Journal of Affective Disorders.

[57] Smith R, Pioro E, Myers K, Sirdofsky M, Goslin K, Meekins G, Pattee G (2017). Enhanced bulbar function in amyotrophic lateral sclerosis: The nuedexta treatment trial. Neurotherapeutics.

[58] Turner MR, Talbot K (2020). Primary lateral sclerosis: Diagnosis and management. Practical Neurology.

[59] Weidner, N., Baumberger, M., Göggelmann, C., Marcus, O., Wittgruber, G., & Wildburger, R. (2020). Thromboembolieprophylaxe bei Querschnittlähmung Entwicklungsstufe S1Stand 1.9.2020 AWMF-Register Nr: 179-015.

[60] Weiss MD, Macklin EA, Simmons Z, Knox AS, Greenblatt DJ, Atassi N, Mexiletine ALS Study Group (2016). A randomized trial of mexiletine in ALS: Safety and effects on muscle cramps and progression. Neurology.

[61] Wirth B, Karakaya M, Kye MJ, Mendoza-Ferreira N (2020). Twenty-five years of spinal muscular atrophy research: From phenotype to genotype to therapy, and what comes next. Annual Review of Genomics and Human Genetics.

[62] Writing Group; Edaravone (MCI-186) ALS 19 Study Group (2017). Safety and efficacy of edaravone in well defined patients with amyotrophic lateral sclerosis: A randomised, double-blind, placebo-controlled trial. Lancet Neurology.

[63] Young CA, Ellis C, Johnson J, Sathasivam S, Pih N (2011). Treatment for sialorrhea (excessive saliva) in people with motor neuron disease/amyotrophic lateral sclerosis. Cochrane Database of Systematic Review.

[64] Zee AA, van Lieshout K, van der Heide M, Janssen L, Janzing HM (2017). Low molecular weight heparin for prevention of venous thromboembolism in patients with lower-limb immobilization. Cochrane Database of Systematic Review.

